# Signal regulatory protein alpha (SIRPα) regulates the homeostasis of CD103^+^CD11b^+^ DCs in the intestinal lamina propria

**DOI:** 10.1002/eji.201444859

**Published:** 2014-10-27

**Authors:** Charlotte L Scott, Zangerle Murray TFP, Katherine S H Beckham, Gillian Douce, Allan McI Mowat

**Affiliations:** 1 Centre for Immunobiology, Institute of Infection, Immunity and Inflammation, College of Veterinary, Medical and Life Sciences, University of GlasgowScotland, UK; 2 VIB Ghent University, Inflammation Research Centre (IRC), Laboratory of ImmunoregulationGhent (Zwijnaarde), Belgium; 3 Department of Respiratory Medicine, Ghent University HospitalGhent, Belgium

**Keywords:** Dendritic cells, Development, Homeostasis, Intestine, SIRPα

## Abstract

Signal regulatory protein alpha (SIRPα/CD172a) is a conserved transmembrane protein thought to play an inhibitory role in immune function by binding the ubiquitous ligand CD47. SIRPα expression has been used to identify dendritic cell subsets across species and here we examined its expression and function on intestinal DCs in mice. Normal mucosa contains four subsets of DCs based on their expression of CD103 and CD11b and three of these express SIRPα. However, loss of SIRPα signaling in mice leads to a selective reduction in the CD103^+^CD11b^+^ subset of DCs in the small intestine, colon, and among migratory DCs in the mesenteric lymph node. In parallel, these mice have reduced numbers of T_H_17 cells in steady-state intestinal mucosa, and a defective T_H_17 response to *Citrobacter* infection. Identical results were obtained in CD47KO mice. DC precursors from SIRPα mutant mice had an enhanced ability to generate CD103^+^CD11b^+^ DCs in vivo, but CD103^+^CD11b^+^ DCs from mutant mice were more prone to die by apoptosis. These data show a previously unappreciated and crucial role for SIRPα in the homeostasis of CD103^+^CD11b^+^ DCs in the intestine, as well as providing further evidence that this subset of DCs is critical for the development of mucosal T_H_17 responses.

## Introduction

The intestinal immune system is exposed to a wide variety of foreign antigens including dietary constituents, commensal microorganisms and pathogens. DCs, the professional antigen presenting cells in the gut, must ensure that the correct kind of T cell is primed so that tolerance or protective immunity is induced appropriately 1,2. We and others have shown recently that four distinct subsets of genuine DCs can be identified in the intestine on the basis of CD103 and CD11b expression 2–6, but the contribution of each subset to the different kinds of intestinal immune responses remains largely unknown.

Signal regulatory protein alpha (SIRPα/CD172a) expression is found on the majority of myeloid cells. However, it is expressed differentially by subsets of DCs, being present on CD11b^+^ DCs in mice, but not on the DCs with cross-presenting activity characterized by expression of CD8α and CD103 6–10. SIRPα is a transmembrane receptor whose cytoplasmic domain contains a tyrosine-based inhibition motif that binds and activates SHP1 and SHP2 phosphatases 9,11,12. The ligand for SIRPα is the ubiquitously expressed CD47 and this interaction is generally believed to have inhibitory effects on immune function, having been implicated in the pathogenesis of a number of models of autoimmunity including experimental autoimmune encephalomyelitis, contact hypersensitivity, and collagen-induced arthritis 13–16.

The SIRPα-CD47 axis has also been implicated in regulating immunity in the gut, although the exact effects and basis for this regulation remain unclear. Thus although CD47KO mice have reduced susceptibility to experimental colitis 17 and decreased intestinal IgA production, they show normal tolerance when administered protein antigens orally 18.

SIRPα mutant mice 19, which have a truncated cytoplasmic domain of the protein and hence cannot signal intracellularly, have a reduction in the proportion of flagellin inducible IL-17- and IFN-γ-producing T cells in the intestinal lamina propria (LP) 20. Although some of these effects have been linked to abnormalities in the SIRPα^+^ (CD11b^+^) subset of “DCs” 18,20, their interpretation is clouded by the fact that these cells were identified only on the basis of expression of CD11c and MHCII. Furthermore it was assumed that CD11b and CD103 defined mutually exclusive subsets of intestinal DCs as they do in other tissues 21. Recently, we and others have shown that most CD11b^+^CD11c^+^MHCII^+^ cells in the intestinal mucosa are resident macrophages rather than the migratory DCs that are needed to drive naïve T-cell priming. In addition, a substantial population of intestinal DCs express both CD103 and CD11b 4,22–24. Here, we have exploited more rigorous identification strategies to reexamine how the SIRPα-CD47 axis regulates intestinal immunity. We show that although SIRPα is expressed by macrophages and three distinct populations of DCs in the gut; the loss of CD47 or SIRPα signaling leads to a selective decrease in CD103^+^CD11b^+^ DCs, together with a decrease in the generation of intestinal T_H_17 cells. The loss of CD103^+^CD11b^+^ DCs in SIRPα mutant mice appears to reflect enhanced susceptibility of these cells to die by apoptosis rather than defective generation from DC progenitors.

## Results

### Loss of SIRPα-CD47 signaling results in a specific reduction in intestinal CD103^+^CD11b^+^ DCs

**Figure 1:**
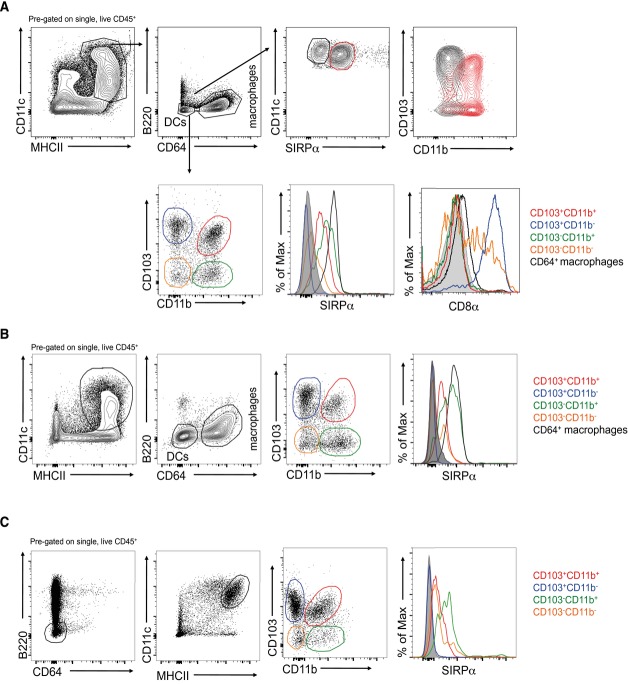
SIRPα expression on intestinal mononuclear phagocytes. (A) Mononuclear phagocytes were identified among live single CD45^+^ cells from enzymatically digested small intestinal lamina propria as CD11c^+^MHCII^+^. Contaminating B cells were excluded on the basis of B220 expression and DCs and macrophages were identified as CD64^−^ or CD64^+^, respectively. Top panels: Expression of CD103 and CD11b by CD11c^+^SIRPα^−^ (black) and CD11c^+^SIRPα^+^ (red) DCs. Bottom panels: Expression of SIRPα and CD8α by DC subsets and CD64^+^ macrophages (isotype controls shown in gray). (B) Expression of SIRPα by mononuclear phagocytes in the colonic LP gated as described in (A). (C) Expression of SIRPα by CD103/CD11b-based subsets of migratory (CD11c^+^MHCII^hi^) DCs in the mesenteric lymph node. (A–C) Plots are from one experiment representative of at least six independent experiments, with *n* = 3–4 mice/experiment.

We first investigated exactly which small intestine lamina propria (SI LP) DC subsets expressed SIRPα, using our recently established gating strategy in which bona fide DCs among mucosal mononuclear phagocytes (MPs) are identified as CD11c^+^MHCII^+^CD64^−^F4/80^−^ 3,4,22,30. On this basis, three SIRPα-expressing subsets of DCs could be observed: CD103^+^CD11b^+^, CD103^−^CD11b^+^, and CD103^−^CD11b^−^ (Fig.1A). The SIRPα^−^ DC population contained both CD103^+^CD11b^−^ and CD103^−^CD11b^−^ subsets, with all CD103^+^CD11b^−^ DCs failing to express SIRPα, whereas the CD103^−^CD11b^−^ population was heterogeneous for SIRPα expression. SIRPα expression was mutually exclusive to that of CD8α, which was found on all CD103^+^CD11b^−^ DCs and on some CD103^−^CD11b^−^ DCs, but not on the CD11b^+^ subsets (Fig.1A). As expected 4,22, all CD11c^+^MHCII^+^CD64^+^F4/80^+^ resident macrophages also expressed SIRPα (Fig.1A). Similar patterns of staining were observed among the mononuclear phagocytes in the colonic LP (Fig.1B) and among migratory DC (CD11c^+^MHCII^hi^) in the mesenteric lymph nodes (Fig.1C).

**Figure 2:**
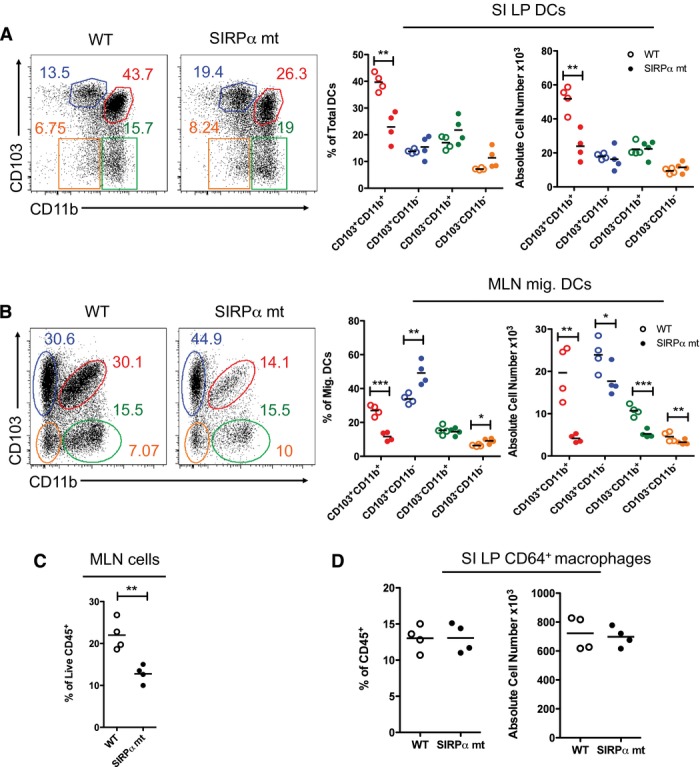
SIRPα signaling controls the homeostasis of CD103^+^CD11b^+^ DCs in vivo. (A) Proportions and absolute numbers of CD103/CD11b-based subsets among live CD45^+^CD11c^+^MHCII^+^CD64^−^B220^−^ DCs from small intestinal LP of SIRPα mutant (mt) (filled circles) and WT (empty circles) mice. (B) Proportions and absolute numbers of CD103/CD11b-based subsets among CD11c^+^MHCII^hi^ migratory DCs in the MLNs of SIRPα mt and WT mice. (C) Frequency of live total CD45^+^ cells in the MLNs of SIRPα mt and WT mice. (D) Proportions and numbers of CD64^+^ macrophages among live CD45^+^CD11c^+^MHCII^+^ cells in small intestinal LP of SIRPα mt and WT mice. Data are from one experiment representative of at least five independent experiments, with *n* = 3/4 per experiment. *p < 0.05, **p < 0.01, ***p < 0.005; Student's *t*-test.

Next, we investigated whether SIRPα played a functional role in intestinal DCs, using mice with a truncated cytoplasmic domain of SIRPα (SIRPα mt) that cannot signal intracellularly 19. These mice had a selective reduction in the proportion and absolute number of CD103^+^CD11b^+^ DCs in the SI (Fig.2A), colon (Supporting Information Fig. 1), and among migratory CD11c^+^MHCII^hi^ DCs in the MLNs (Fig.2B). Although the absolute numbers of all migratory DC subsets were reduced in the MLNs of SIRPα mt mice, this reflected a global reduction in cellularity and only the CD103^+^CD11b^+^ DCs showed a proportional defect in mutant MLNs (Fig.2C). The other SIRPα expressing intestinal MPs including macrophages, were unaffected by the loss of SIRPα signaling in either the SI or colon (Fig.2D and Supporting Information Fig. 1C).

### CD47KO mice phenocopy the DC defect in SIRPα mt mice

**Figure 3:**
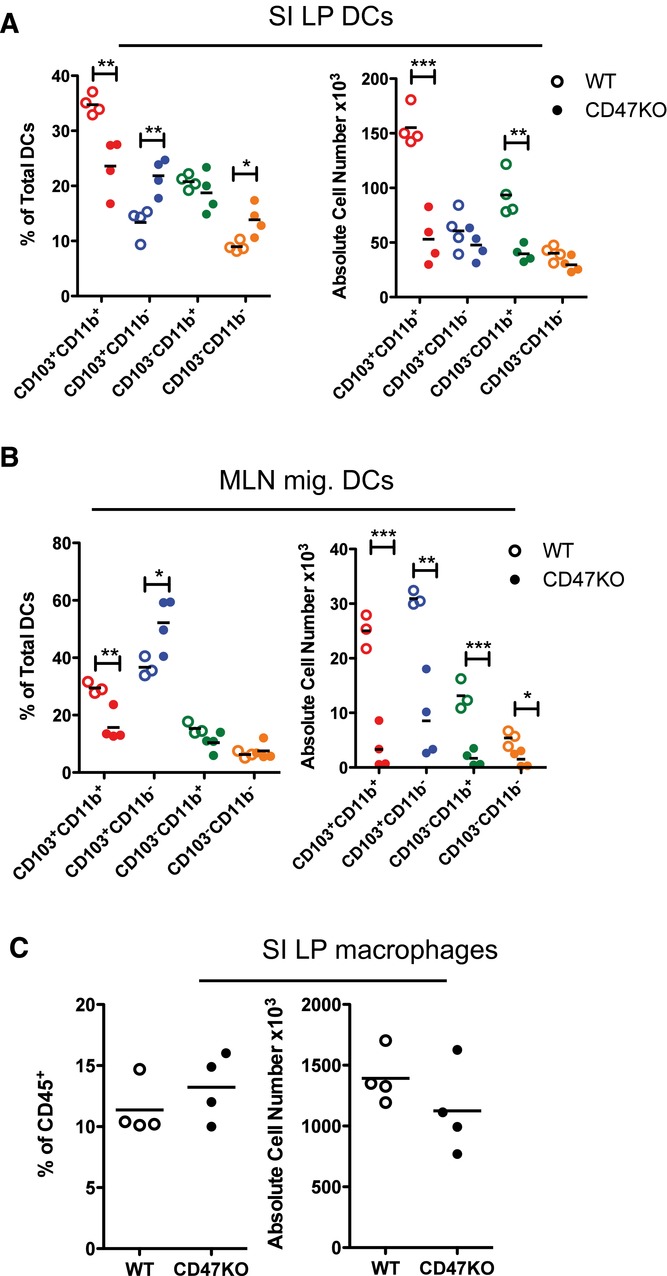
CD47KO mice phenocopy the intestinal DC defect in SIRPα mt mice. (A) Proportions and absolute numbers of CD103/CD11b-based subsets among live CD45^+^CD11c^+^MHCII^+^CD64^−^B220^−^ DCs from small intestinal LP of CD47KO (filled circles) and WT (empty circles) mice. (B) Proportions and absolute numbers of CD103/CD11b-based subsets among CD11c^+^MHCII^hi^ migratory DCs in the MLNs of CD47KO and WT mice. (C) Proportions and numbers of CD64^+^ macrophages among live CD45^+^CD11c^+^MHCII^+^ cells in small intestinal LP of SIRPα mt and WT mice. Data are from one experiment representative of at least three independent experiments, with *n* = 3/4 per experiment. *p < 0.05, **p < 0.01, ***p < 0.005; Student's *t*-test.

CD47KO mice had a selective and equivalent reduction in CD103^+^CD11b^+^ DCs in both the SI LP and migratory compartment of the MLNs (Fig.3A and B), as well as normal proportions and numbers of mucosal macrophages (Fig.3C).

### Reduction in CD103^+^CD11b^+^ DCs correlates with a selective defect in intestinal T_H_17 cells

**Figure 4:**
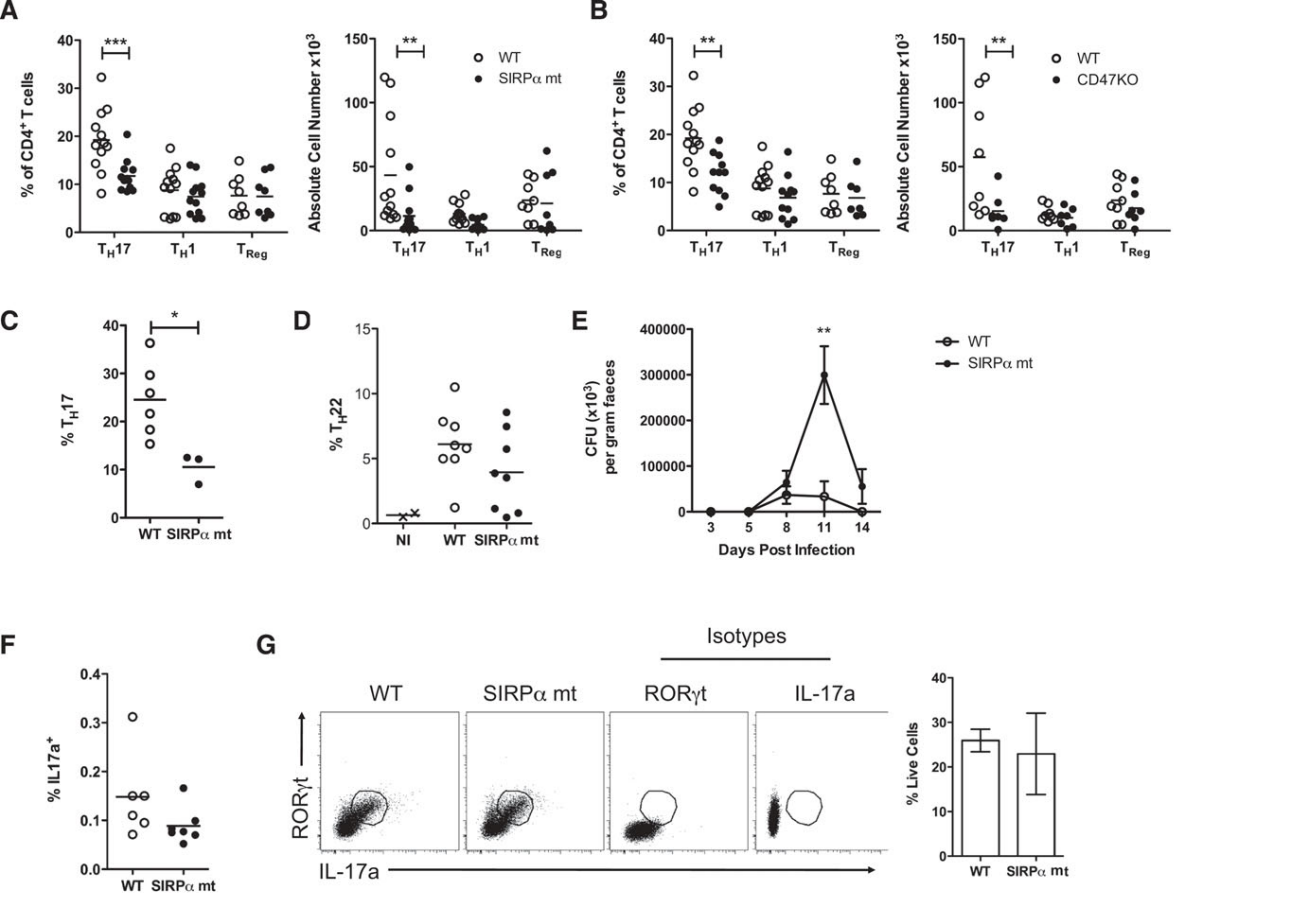
Defects in CD103^+^CD11b^+^ intestinal DCs correlate with reduced T_H_17 cells in LP. (A, B) Proportions and absolute numbers of cells staining intracellularly for IL-17a, FoxP3, and IFN-γ among total live CD4^+^ T cells in the small intestinal LP of SIRPα mt mice (A) and CD47KO mice (B) (filled circles), together with WT controls (empty circles). (A and B) Data are pooled from 2–3 independent experiments, with *n* = 4 per experiment. (C) Mice were infected orally with 1 × 10^9^ CFU of *C. rodentium* and the numbers of IL-17a producing CD4^+^ T cells in MLNs assessed on day 8 of infection by intracellular cytokine staining. Results show proportion of T_H_17 cells as a percentage of total CD4^+^ T cells in the MLN of SIRPα mt and WT mice. Data are from one experiment representative of two independent experiments, with *n* = 3–6 mice per experiment. (D) IL-22 producing CD4^+^ T cells in the colonic LP of SIRPα mt (filled circles) and WT (empty circles) mice 8 days after infection with *C. rodentium* as assessed by intracellular cytokine staining. Results show the proportions of IL-22 producing cells as a percentage of total CD4^+^ T cells. Data are from a single experiment with *n* = 2–8 mice/group. NI (x) represents noninfected WT controls. (E) Course of infection with *C. rodentium* in WT and SIRPα mt mice. The data are shown as mean ± SD (CFU × 10^3^/g feces) from ten mice/group and are from one experiment representative of two experiments. (F) CD103^−^CD11b^+^ DCs (3 × 10^4^) were FACS-purified from the SI LP of WT and SIRPα mt mice, pulsed with 2 mg/mL OVA and cocultured with FACS-purified naïve (CD62L^+^CD25^−^) CD4^+^ OVA-specific OTII transgenic T cells for 4 days before being assessed for IL-17a production by intracellular cytokine staining. Data are pooled from three independent experiments. (G) FACS-purified naive CD62L^+^CD25^−^CD4^+^ T cells from the MLN of SIRPα mt or WT mice were cultured for 4 days on plates coated with anti-CD3 and anti-CD28, together with anti-IFN-γ, anti-IL-4, anti-IL-2, IL-6, IL-23, IL-1β, and TGF-β. RORγt and IL-17a expression were then assessed by intracellular staining. Data are shown as means ± SD pooled from two independent experiments, with *n* = 6-7 per group. *p < 0.05, **p < 0.01; Student's *t*-test

As CD103^+^CD11b^+^ DCs have recently been implicated in the homeostasis of mucosal T_H_17 cells 5,6,13,26–29, we next examined the CD4^+^ T-cell compartment in the small intestinal LP of steady-state SIRPα mt and CD47KO animals. Both SIRPα mt and CD47KO mice showed an approximately 50% reduction in IL-17-producing T_H_17 cells compared with WT LP, whereas the numbers of FoxP3^+^ Treg and IFN-γ^+^ T_H_1 cells were unaffected (Fig.4A and B, and Supporting Information Fig. 2A). In addition, SI LP CD4^+^ T cells FACS-purified from SIRPα mt mice had a trend toward reduced il22 mRNA expression (Supporting Information Fig. 2B). During infection by the intestinal pathogen *Citrobacter rodentium*, SIRPα mt mice also showed defective induction of T_H_17 cells in the MLNs, as well as a trend toward reduced proportions of CD4^+^ T cells producing IL-22 in the colonic LP, where there was a ∼40% reduction compared with the levels in WT colon (Fig.4C and D, and Supporting Information Fig. 2C). These changes in T-cell differentiation were associated with delayed clearance of the pathogen (Fig.4E). Importantly, these differences were not due to impaired IL-22 production by type 3 innate lymphoid cells (ILC3s) (Supporting Information Fig. 2D).

As we have recently shown that CD103^−^CD11b^+^ DCs from the intestine are the main inducers of T_H_17 differentiation in vitro 3,30, we assessed whether a functional defect in this population could account for the impaired T_H_17 priming in SIRPα mt mice, despite the normal numbers of this subset. However, CD103^−^CD11b^+^ DCs from the SI LP of SIRPα mt mice were equally capable of inducing T_H_17 responses as their WT counterparts following ex vivo coculture with naïve CD4^+^ OTII T cells (Fig.4F). The reduced T_H_17 cell generation in vivo was also not due to an intrinsic defect in SIRPα mt T cells, as naïve CD4^+^ T cells from SIRPα mt MLNs could be polarized in vitro to express RORγt and IL17a at levels equivalent to WT MLN CD4^+^ T cells (Fig.4G).

In contrast to this defect in T_H_17 cell generation, regulatory T cell dependent mechanisms appeared to be normal in the absence of SIRPα signaling. Thus there were normal numbers and proportions of FoxP3^+^ Treg cells in the SIRPα mt SI and colon (Fig.4A and B, and Supporting Information Fig. 2A and E), although a slight reduction was observed in the MLNs (Supporting Information Fig. 2F). In addition these mice developed tolerance of systemic delayed type hypersensitivity responses normally when fed OVA before parenteral challenge with antigen in CFA (Supporting Information Fig. 2G).

### Development of CD103^+^CD11b^+^ DC from precursors is enhanced by the loss of SIRPα-CD47 signaling

**Figure 5:**
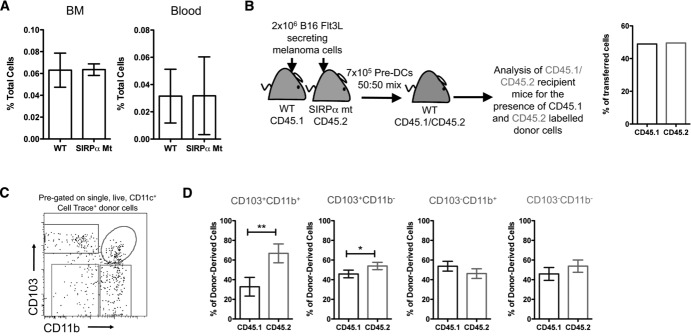
Loss of functional SIRPα signal confers a selective advantage in the generation of CD103^+^CD11b^+^ DCs from pre-DCs. (A) Proportions of Lin^−^CD11c^int^SIRPα^int^CD135^+^ pre-DCs in the BM and blood of WT and SIRPα mt mice. Data are shown as% of total cells ± 1 SD and are from one experiment representative of 2–5 independent experiments with *n* = 3–4 per group per experiment. (B) Lin^−^CD11c^int^SIRPα^int^CD135^+^ pre-DCs were FACS sorted from the BM of CD45.1^+^ WT and CD45.2^+^ SIRPα mt mice ten days after subcutaneous injection of 2 × 10^6^ Flt3L secreting B16 cells, labeled with CellTrace violet dye, mixed in a 50:50 ratio and 7 × 10^5^ cells were transferred i.v. into resting CD45.1^+^/CD45.2^+^ WT recipients. (C) Five days later, CellTrace^+^ total donor cells were identified in the SI LP of recipient mice and examined for CD103 and CD11b expression. (D) Donor-derived DCs were then examined for CD45.1 and CD45.2 expression to assess their origin from WT versus SIRPα mt precursors. The data are shown as the mean ± SD (*n* = 4) and are pooled from two independent experiments. *p < 0.05, **p < 0.01, Student's *t-*test.

As CD103^+^CD11b^+^ intestinal DCs are the progeny of DC-committed progenitors that express SIRPα 23,25,30, we explored whether defective SIRPα signaling might affect the generation of these DCs. Normal numbers of pre-DCs were present in the BM and blood of SIRPα mt mice 31,32 (Fig.5A). To study their ability to generate intestinal DCs in vivo, CD45.1^+^ WT and CD45.2^+^ SIRPα mt pre-DCs were transferred in a 50:50 ratio into CD45.1^+^/CD45.2^+^ WT recipient mice (Fig.5B). The mature progeny were then identified in the SI LP 5 days after transfer (Fig.5C), a time we had found optimal for the development of DCs in the gut in this system (data not shown). Unexpectedly, SIRPα mt pre-DCs appeared to be more effective at generating CD103^+^CD11b^+^ DCs in LP than their WT counterparts, as well as being somewhat better at generating SIRPα^−^CD103^+^CD11b^−^ DCs (Fig.5D). SIRPα mt and WT pre-DCs had equal abilities to generate the two CD103^−^ DC populations, both of which express SIRPα (Fig.5D).

### Increased apoptosis of CD103^+^CD11b^+^ DCs in the absence of a functional SIRPα signal

**Figure 6:**
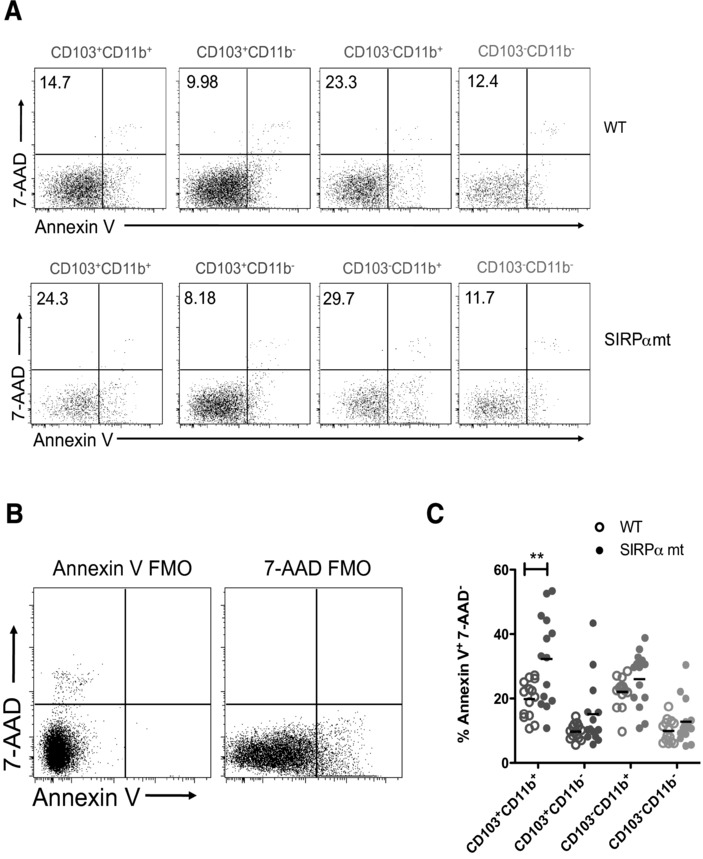
Increased apoptosis of CD103^+^CD11b^+^ DCs in the absence of a functional SIRPα signal. DC subsets among CD11c^+^MHCII^hi^ migratory DCs in whole MLNs isolates from WT and SIRPα mt mice were stained for Annexin V and 7-AAD. (A) Representative plots showing Annexin V and 7-AAD staining by CD103/CD11b DC subsets among CD11c^+^MHCII^hi^ migratory DCs. Numbers represent proportions of apoptotic (Annexin V^+^ 7-AAD^−^) cells in each subset. (B) Representative plot showing Annexin V and 7-AAD staining in fluorescence minus one controls used to set gates among total migratory (CD11c^+^ MHCII^hi^) DCs. (A and B) Plots are from one representative of four experiments, with 3–4 mice/group/experiment. (C) Proportions of apoptotic (Annexin V^+^ 7-AAD^−^) cells as a percentage of each DC subset in WT and SIRPα mt MLNs. Data are pooled from four independent experiments, with *n* = 3–4 mice/group/experiment. **p < 0.01; Student's *t*-test.

The selective advantage of SIRPα mt pre-DCs in generating CD103^+^CD11b^+^ DCs, despite the marked deficiency in this subset seen in SIRPα mice, suggested that these DCs might be compromised by the lack of SIRPα signaling later in their life. To explore this, we compared the apoptosis of intestinal DC subsets in SIRPα mt and WT mice. Annexin V staining showed that migratory CD103^+^CD11b^+^ DCs were more prone to apoptosis in SIRPα than in WT MLNs. In contrast, no differences in Annexin V staining were noted among the other DC populations (Fig.6). Thus the SIRPα/CD47 axis appears to be important for promoting the survival of intestinal CD103^+^CD11b^+^ DCs.

## Discussion

Here, we have exploited recent advances in characterizing intestinal DCs and their subsets to explore the significance of SIRPα expression on these cells. We demonstrate that three of the four subsets of bona fide DCs, that we and others have identified in the mouse intestine 3–5,30 express SIRPα, these being CD103^+^CD11b^+^, CD103^−^CD11b^+^, and CD103^−^CD11b^−^. The remaining CD103^+^CD11b^−^ DCs are uniformly SIRPα^−^ and comprise the CD8α^+^DNGR-1^+^XCR1^+^ population responsible for cross-presentation 33. Some CD103^−^CD11b^−^ DCs also fail to express SIRPα, consistent with previous findings that this subset is phenotypically heterogeneous and its functions remain to be elucidated 3.

Despite its widespread expression, loss of SIRPα signaling in SIRPα mt mice caused a selective reduction in CD103^+^CD11b^+^ DCs in the LP of the entire intestinal tract and among migratory DCs in MLNs. The other subsets of SIRPα^+^ DC were unaffected, as were CD64^+^ macrophages, which are uniformly SIRPα^+^. Identical results were obtained in mice lacking the ligand for SIRPα, CD47, extending a previous study that found reduced CD103^+^CD11b^+^ LP DCs in CD47KO mice, but in which the other DC populations were not examined 18. Other groups also found reduced numbers of CD11b^+^ “DCs” in the LP of SIRPα mt and CD47KO mice, but these were reported to lack CD103 and the cells analyzed were total CD11c^+^CD11b^+^ MPs 17,20. As we show here, this population is highly heterogeneous, containing macrophages, CD103^−^CD11b^+^ DCs and CD103^+^CD11b^+^ DCs, which can only be distinguished by multiparameter analysis. As CD103^+^CD11b^+^ DCs are a relatively minor part of this overall population, they could easily have been overlooked in earlier studies.

As has been reported previously 13,34–36, the reduction in CD103^+^CD11b^+^ DCs which we observed in the intestine of SIRPα mt and CD47KO mice was accompanied by a defect in CD11b expressing DCs in other tissues such as the spleen. However, the phenotypically identical CD103^−^CD11b^+^ DC subset in the intestine was not affected by the SIRPα mutation. CD103 expression on CD11b^+^ (SIRPα^+^) DCs is unique to the intestine and appears to reflect tissue specific "conditioning" by that environment 2. Similarly concordant defects in CD11b^+^ splenic DCs and in the CD103^+^CD11b^+^ subset in LP are also present in mice with IRF4 and Notch2 deficiency targeted to CD11c^+^ cells, but the effects on CD103^−^CD11b^+^ DCs in the intestine of these mice remain to be investigated 5,6,26,37. Together, our results could suggest a model in which mucosal CD103^−^CD11b^+^ DCs are less mature than either the CD103^+^CD11b^+^ DCs in intestine or CD11b^+^ DC in the spleen, and that SIRPα signaling is essential for their full differentiation. We are currently examining this idea in more detail.

The loss of CD103^+^CD11b^+^ intestinal DCs in SIRPα mt and CD47KO mice was accompanied by a selective reduction in IL-17/IL-22 producing CD4^+^ T cells in the steady-state intestinal LP, whereas T_H_1 cells and FoxP3^+^ Treg cells were unaffected. Similar defects in T_H_17/22 generation were found during infection with *C. rodentium* in SIRPα mt mice, which also showed delayed clearance of the organism. Protective immunity in this infection requires IL-22 and IL-17, produced by different cells in two distinct phases. Early in infection, these cytokines are derived from ILC3s, whereas CD4^+^ T cells are needed for the later stage in which the pathogen is cleared 38. The role of CD103^+^CD11b^+^ DCs in this infection has been controversial, as although one report suggested that they were required to drive the early IL-22 production by ILC3s 37, others found that CD103^+^CD11b^+^ DCs were not required for clearance of the organism 27. Here, we found that the delayed clearance in SIRPα mt mice only became apparent at the later stages of infection and this correlated with fewer T_H_17 and T_H_22 cells in the MLNs and colonic LP. In contrast, we could not find any defect in IL-22 production by ILC3 in SIRPα mt mice. For these reasons we conclude that the enhanced susceptibility of these mice to *C. rodentium* infection reflects defective priming of adaptive effector T cells by CD103^+^CD11b^+^ DCs rather than an effect on ILC3s.

Our findings of reduced generation of intestinal T_H_17 cells in SIRPα mt mice are consistent with other disease models in these mice, including EAE, contact hypersensitivity and collagen-induced arthritis 13,14,16. Recent studies in other mouse models have also confirmed a specific link between reduced numbers of CD103^+^CD11b^+^ DCs and fewer T_H_17 cells in the gut, perhaps reflecting reduced production of polarizing cytokines such as IL-6 or IL-23 5,6,26,27,37. However, the exact mechanisms underlying the connection remain to be elucidated and it should be noted that our recent studies show that CD103^−^CD11b^+^ DCs are the most effective inducers of T_H_17 cell differentiation when DC subsets from intestinal LP or lymph are assessed using naïve antigen-specific CD4^+^ T cells in vitro 3,30. The explanation for these apparently discordant results is unknown, but could indicate that the CD103^+^ and CD103^−^ subsets of CD11b^+^ DCs may represent different stages in the same developmental pathway, or they may need to interact together in vivo to generate T_H_17 cells. Alternatively the subsets may play separate roles in the induction and subsequent maintenance of T_H_17 cells in vivo. This latter idea could be consistent with work suggesting that the effects of CD103^+^CD11b^+^ DCs on the homeostasis of T_H_17 cells in the intestine may not require presentation of cognate antigen 27. Furthermore, recent studies show that the induction of T_H_17 cells in the intestine by Segmented Filamentous Bacteria involves two processes, one of which requires uptake via secondary lymphoid organs and presentation of specific antigen by DCs; a further population of segmented filamentous bacteria-dependent T_H_17 cells is independent of these events 39,40. More work is needed to define better the link between different DC subsets and T_H_17 cell homeostasis in the intestine.

The loss of SIRPα signaling did not affect the numbers of FoxP3^+^ Treg in the steady-state LP, and SIRPα mt mice developed systemic tolerance normally after feeding protein, a phenomenon that is dependent on Treg cells 1. These results are consistent with previous studies showing normal oral tolerance in CD47KO mice 18 and with other recent work showing normal numbers of FoxP3^+^ Treg cells when CD103^+^CD11b^+^ DCs are lacking 2,27. Thus original assumptions that intestinal CD103^+^ DCs were intrinsically tolerogenic need to be reassessed in the light of newer insights into their heterogeneity and function 41–44.

Previous studies have suggested that defective numbers of CD11b^+^ DCs in secondary lymphoid organs of SIRPα mt or CD47KO mice might reflect reduced migration via afferent lymphatics 35,36,45. However, this is unlikely to account for our findings, as we observed a similar defect CD103^+^CD11b^+^ DCs in both the mucosa and draining MLNs. Although the committed precursors of DCs express SIRPα 31, their numbers were not altered in the BM or blood of SIRPα mt mice. Indeed we found that these pre-DCs actually appeared to be more efficient at generating CD103^+^CD11b^+^ DCs in the mucosa compared with WT pre-DCs. This indication that SIRPα may normally function as a checkpoint in the development of a specific subset of intestinal DCs contradicts previous findings in the spleen where pre-DCs from SIRPα mt mice were found to have a reduced ability to generate CD11b^+^ DC compared with WT pre-DCs 34. This could reflect an intrinsic difference in ontogeny between CD11b^+^ DCs in systemic lymphoid tissues and intestinal CD103^+^CD11b^+^ DCs; indeed the latter DCs are not found elsewhere in the body. An alternative explanation may be that we analyzed donor cells 5 days after transfer, whereas Saito et al did not examine the fate of pre-DCs in the spleen until 8 days after transfer 34. Interestingly, these groups also found that the progeny of SIRPα mt pre-DCs had an unusually short half-life in vivo and indeed, we did not find enhanced generation of CD103^+^CD11b^+^ by SIRPα mt pre-DCs when the intestine was examined 7 days after transfer. Thus, this earlier study may have missed the accelerated development that we observed.

The idea that CD103^+^CD11b^+^ DCs in SIRPα mt mice are compromised in their survival was supported by their increased susceptibility to apoptosis in the MLNs. Although the lengthy enzymatic digestion needed to isolate LP DCs precluded analysis of apoptosis in these cells, all CD103^+^CD11b^+^ DCs in the MLNs are within the “migratory” gate, indicating they have come from the mucosa 3,46. Thus reduced survival is likely to be an intrinsic property of this subset that resides in LP as part of its life cycle. Whether these cells actually die in the mucosa itself, or their survival defect only becomes apparent once they have migrated to the MLNs remains to be determined.

Overall our results demonstrate a previously unappreciated role for SIRPα in the homeostasis of CD103^+^CD11b^+^ DCs in the intestine. We propose that this subset of DCs develops more rapidly in the absence of SIRPα and is then more susceptible to activation and subsequent death. SIRPα may therefore normally act as a brake on these processes via its ability to inhibit signaling pathways by binding SHP1 phosphatase 12. However, an alternative explanation could come from the finding that SIRPα can also promote survival pathways in other cell types via SHP2-mediated induction of MAPK, PI3 kinase, and NF-κB 47. Loss of SIRPα signaling could therefore compromise DC survival via this mechanism. As our own and other recent work indicates that SIRPα expressing DCs are also present in substantial numbers in human intestine 6,10,30 elucidating the mechanisms of SIRPα mediated control of their development and functions could have important clinical implications.

## Materials and methods

### Mice

Wild-type C57BL/6 (B6) mice were purchased from Harlan Olac (Bicester, UK). SIRPα mt mice 19 were obtained from Dr. PA Oldenberg (Umea University, Sweden) with kind permission from T. Matozaki (University of Tokyo, Japan). CD47KO mice 48 were purchased from Jackson Laboratories (Maine, USA). All strains were backcrossed for at least nine generations on to the B6 background and were maintained under specific pathogen free conditions at the University of Glasgow animal facilities, before being used between 6 and 12 weeks of age. Animal experiments were performed in accordance with UK Home Office guidelines.

### Murine cell isolation

Lamina propria cells were obtained from murine intestines by enzymatic digestion as previously described 3,49. Cells were isolated from mesenteric lymph nodes by enzymatic digestion with 1 mg/mL collagenase D (Roche) in calcium magnesium free Hank's balanced salt solution (Gibco, Invitrogen) for 45 minutes. After isolation, cells were passed through a 100 μm and then a 40 μm filter before use (Corning).

### Flow cytometric analysis and sorting of cells

Cells were stained at 4°C in the dark, as previously described in 49. For intracellular cytokine staining, whole LP digests were incubated for 4.5 h with 1 × Cell stimulation cocktail (eBioscience) before fixation and permeabilisation. In all analyses, following doublet exclusion, live cells were identified using 7-AAD (Biolegend) or fixable viability dye (eBioscience). Data were acquired on an LSR II or FACSAria I (BD Biosciences) and analyzed using FlowJo software (Tree Star Inc).

### T-cell polarization in vitro

Ultra low adherence 24-well plates were coated with 1.5 μg/mL anti-CD3 and 1.5 μg/mL anti-CD28 (BD Biosciences) in calcium magnesium free PBS for 6 h at 4°C. After washing the plates, 8 × 10^5^ FACS-purified naïve CD4^+^CD62L^hi^ CD25^−^ T cells from MLNs were added in 1 mL complete RPMI supplemented with 10 μg/mL anti-IFN-γ, 10 μg/mL anti-IL-4, 10 μg/mL anti-IL-2, 20 ng/mL IL-6, 20 ng/mL IL-23, 20 ng/mL IL-1β (all BD Biosciences), and 2.5 ng/mL recombinant human TGF-β (Peprotech). Cells were incubated at 37°C with 5% CO_2_ for 4 days and supplemented with 500 μL complete RPMI on day 3. On day 4 cells were harvested and cultured with cell stimulation cocktail for 4.5 h at 37°C with 5% CO_2_. The cells were then harvested and stained for intracellular IL-17a and RORγt.

### DC: T cell cocultures

CD103^−^CD11b^+^ DCs (3 × 10^4^) were FACS-purified from the SI LP and pulsed with 2 mg/mL OVA for 2 h. Cells were then washed extensively and cocultured for 4 days with 1 × 10^5^ CFSE-labeled naïve CD4^+^ OVA-specific Transgenic OTII T cells (sorted as CD62L^hi^, CD25^−^). Following coculture, T cells were restimulated for 4.5 h with 1× cell stimulation cocktail (eBiosciences) and IL-17a production was assessed as described above.

### Adoptive transfer of pre-DCs

To expand pre-DCs, CD45.1^+^ WT or CD45.2^+^ SIRPα mt mice were injected with 2 × 10^6^ flt3L secreting B16 tumor cells subcutaneously (a kind gift from Dr. Oliver Pabst, Hannover, Germany) and 10–14 days later, BM was isolated and RBCs lysed (Stem Cell Technologies). Cells were labeled with eFluor450 CellTrace Violet proliferation dye (eBioscience) and pre-DCs were identified as Lin^−^ (CD3, CD19, B220, CD49b, MHCII, and CD11b), CD11c^int^ SIRPα^int^CD135^+^ cells as previously reported 30. A total of 3.5 × 10^5^ FACS sorted pre-DCs were injected into unmanipulated CD45.1^+^/CD45.2^+^ recipients in a 50:50 mixture. Five days later recipient mice were examined for donor cells.

### Assessment of apoptosis

Apoptosis was assessed on MLN cells by staining for Annexin V (BD Biosciences) in conjunction with 7-AAD according to the manufacturer's guidelines and analyzed by flow cytometry.

### *C. rodentium* infection

*C. rodentium* (ATCC 51459) was cultured with aeration in DMEM to log phase (OD_650_ = 1.0) before concentration by centrifugation. WT and SIRPα mt mice were inoculated with 1 × 10^9^ *C. rodentium* organisms by oral gavage and the level of infection was quantified by colony counts in feces. On day 7, mice were sacrificed and IL-17a producing cells were identified in MLNs following 4.5 h restimulation with 1× Cell stimulation cocktail as described above.

### Statistical analysis

Results are presented as means ±1 SD unless otherwise stated and groups were compared using a Student's *t*-test, or for multiple groups, a one-way ANOVA followed by a Bonferroni posttest using Prism Software (GraphPad Software, Inc.).

## Abbreviations:

HAO: heat aggregated OVA
ILC3: type 3 innate lymphoid cell
LP: lamina propria
MP: mononuclear phagocyte
mt: mutant
SI: small intestine
SIRPα: signal regulatory protein α

## Supporting Information

As a service to our authors and readers, this journal provides supporting information supplied by the authors. Such materials are peer reviewed and may be re-organized for online delivery, but are not copy-edited or typeset. Technical support issues arising from supporting information (other than missing files) should be addressed to the authors.

**Figure S1**. Effect of SIRPα mutation on mononuclear phagocytes in the colonic lamina propria. **Figure S2**. Reduced population of CD103^+^CD11b^+^ DCs does not impact TReg cells in the colonic LP or affect the induction of oral tolerance.

## References

[1] Pabst O, Mowat AM (2012). Oral tolerance to food protein. Mucosal Immunol.

[2] Persson EK, Scott CL, Mowat AM, Agace WW (2013). Dendritic cell subsets in the intestinal lamina propria: ontogeny and function. Eur. J. Immunol.

[3] Cerovic V, Houston SA, Scott CL, Aumeunier A, Yrlid U, Mowat AM, Milling SWF (2013). Intestinal CD103(-) dendritic cells migrate in lymph and prime effector T cells. Mucosal Immunol.

[4] Tamoutounour S, Henri S, Lelouard H, de Bovis B, de Haar C, van der Woude CJ, Woltman AM (2012). CD64 distinguishes macrophages from dendritic cells in the gut and reveals the Th1-inducing role of mesenteric lymph node macrophages during colitis. Eur. J. Immunol.

[5] Schlitzer A, McGovern N, Teo P, Zelante T, Atarashi K, Low D, Ho AWS (2013). IRF4 transcription factor-dependent CD11b(+) dendritic cells in human and mouse control mucosal IL-17 cytokine responses. Immunity.

[6] Persson EK, Uronen-Hansson H, Semmrich M, Rivollier A, Hägerbrand K, Marsal J, Gudjonsson S (2013). IRF4 transcription-factor-dependent CD103(+)CD11b(+) dendritic cells drive mucosal T helper 17 cell differentiation. Immunity.

[7] Milling SWF, Jenkins CD, Yrlid U, Cerovic V, Edmond H, McDonald V, Nassar M (2009). Steady-state migrating intestinal dendritic cells induce potent inflammatory responses in naive CD4+ T cells. Mucosal Immunol.

[8] Bimczok D, Sowa EN, Faber-Zuschratter H, Pabst R, Rothkötter H-J (2005). Site-specific expression of CD11b and SIRPalpha (CD172a) on dendritic cells: implications for their migration patterns in the gut immune system. Eur. J. Immunol.

[9] Barclay AN, Brown MH (2006). The SIRP family of receptors and immune regulation. Nat. Rev. Immunol.

[10] Watchmaker PB, Lahl K, Lee M, Baumjohann D, Morton J, Kim SJ, Zeng R (2014). Comparative transcriptional and functional profiling defines conserved programs of intestinal DC differentiation in humans and mice. Nat. Immunol.

[11] Barclay A (2009). Signal regulatory protein alpha (SIRP[alpha])/CD47 interaction and function. Curr. Opin. Immunol.

[12] Matozaki T, Murata Y, Okazawa H, Ohnishi H (2009). Functions and molecular mechanisms of the CD47-SIRPalpha signalling pathway. Trends Cell Biol.

[13] Tomizawa T, Kaneko Y, Saito Y, Ohnishi H, Okajo J, Okuzawa C, Ishikawa-Sekigami T (2007). Resistance to experimental autoimmune encephalomyelitis and impaired T cell priming by dendritic cells in Src homology 2 domain-containing protein tyrosine phosphatase substrate-1 mutant mice. J. Immunol.

[14] Okuzawa C, Kaneko Y, Murata Y, Miyake A, Saito Y, Okajo J, Tomizawa T (2008). Resistance to collagen-induced arthritis in SHPS-1 mutant mice. Biochem. Biophys. Res. Commun.

[15] Fukunaga A, Nagai H, Yu X, Oniki S, Okazawa H, Motegi S-I, Suzuki R (2006). Src homology 2 domain-containing protein tyrosine phosphatase substrate 1 regulates the induction of Langerhans cell maturation. Eur. J. Immunol.

[16] Motegi S-I, Okazawa H, Murata Y, Kanazawa Y, Saito Y, Kobayashi H, Ohnishi H (2008). Essential roles of SHPS-1 in induction of contact hypersensitivity of skin. Immunol. Lett.

[17] Fortin G, Raymond M, Van VQ, Rubio M, Gautier P, Sarfati M, Franchimont D (2009). A role for CD47 in the development of experimental colitis mediated by SIRPalpha+CD103- dendritic cells. J. Exp. Med.

[18] Westlund J, Livingston M, Fahlén-Yrlid L, Oldenborg P-A, Yrlid U (2011). CD47-deficient mice have decreased production of intestinal IgA following oral immunization but a maintained capacity to induce oral tolerance. Immunology.

[19] Inagaki K, Yamao T, Noguchi T, Matozaki T, Fukunaga K, Takada T, Hosooka T (2000). SHPS-1 regulates integrin-mediated cytoskeletal reorganization and cell motility. EMBO J.

[20] Kanazawa Y, Saito Y, Supriatna Y, Tezuka H, Kotani T, Murata Y, Okazawa H (2010). Role of SIRPα in regulation of mucosal immunity in the intestine. Genes Cells.

[21] del Rio M-L, Bernhardt G, Rodriguez-Barbosa J-I, Förster R (2010). Development and functional specialization of CD103+ dendritic cells. Immunol. Rev.

[22] Bain CC, Scott CL, Uronen-Hansson H, Gudjonsson S, Jansson O, Grip O, Guilliams M (2013). Resident and pro-inflammatory macrophages in the colon represent alternative context-dependent fates of the same Ly6Chi monocyte precursors. Mucosal Immunol.

[23] Bogunovic M, Ginhoux F, Helft J, Shang L, Hashimoto D, Greter M, Liu K (2009). Origin of the lamina propria dendritic cell network. Immunity.

[24] Varol C, Vallon-Eberhard A, Elinav E, Aychek T, Shapira Y, Luche H, Fehling HJ (2009). Intestinal lamina propria dendritic cell subsets have different origin and functions. Immunity.

[25] Schraml BU, van Blijswijk J, Zelenay S, Whitney PG, Filby A, Acton SE, Rogers NC (2013). Genetic tracing via DNGR-1 expression history defines dendritic cells as a hematopoietic lineage. Cell.

[26] Lewis KL, Caton ML, Bogunovic M, Greter M, Grajkowska LT, Ng D, Klinakis A (2011). Notch2 receptor signaling controls functional differentiation of dendritic cells in the spleen and intestine. Immunity.

[27] Welty NE, Staley C, Ghilardi N, Sadowsky MJ, Igyártó BZ, Kaplan DH (2013). Intestinal lamina propria dendritic cells maintain T cell homeostasis but do not affect commensalism. J. Exp. Med.

[28] Latour S, Tanaka H, Demeure C, Mateo V, Rubio M, Brown EJ, Maliszewski C (2001). Bidirectional negative regulation of human T and dendritic cells by CD47 and its cognate receptor signal-regulator protein-alpha: down-regulation of IL-12 responsiveness and inhibition of dendritic cell activation. J. Immunol.

[29] Waclavicek M, Majdic O, Stulnig T, Berger M, Baumruker T, Knapp W, Pickl WF (1997). T cell stimulation via CD47: agonistic and antagonistic effects of CD47 monoclonal antibody 1/1A4. J. Immunol.

[30] Scott CL, Bain CC, Wright PB, Sichien D, Kotarsky K, Persson EK, Luda K (2014). CCR2^+^CD103^−^intestinal dendritic cells develop from DC committed progenitors and induce interleukin 17 production by T cells. Mucosal Immunol.

[31] Liu K, Victora GD, Schwickert TA, Guermonprez P, Meredith MM, Yao K, Chu F-F (2009). In vivo analysis of dendritic cell development and homeostasis. Science.

[32] Naik SH, Sathe P, Park H-Y, Metcalf D, Proietto AI, Dakic A, Carotta S (2007). Development of plasmacytoid and conventional dendritic cell subtypes from single precursor cells derived in vitro and in vivo. Nat. Immunol.

[33] Cerovic V, Houston SA, Westlund J, Utriainen L, Davison E, Scott CL, Bain CC (2014). Lymph borne CD8a^+^DCs are uniquely able to cross-prime CD8^+^ T cells with antigen acquired from intestinal epithelial cells. Mucosal Immunol.

[34] Saito Y, Iwamura H, Kaneko T, Ohnishi H, Murata Y, Okazawa H, Kanazawa Y (2010). Regulation by SIRPα of dendritic cell homeostasis in lymphoid tissues. Blood.

[35] Iwamura H, Saito Y, Sato-Hashimoto M, Ohnishi H, Murata Y, Okazawa H, Kanazawa Y (2011). Essential roles of SIRPα in homeostatic regulation of skin dendritic cells. Immunol. Lett.

[36] Raymond M, Rubio M, Fortin G, Shalaby KH, Hammad H, Lambrecht BN, Sarfati M (2009). Selective control of SIRP-alpha-positive airway dendritic cell trafficking through CD47 is critical for the development of T(H)2-mediated allergic inflammation. J. Allergy Clin. Immunol.

[37] Satpathy AT, Briseño CG, Lee JS, Ng D, Manieri NA, KC W, Wu X (2013). Notch2-dependent classical dendritic cells orchestrate intestinal immunity to attaching-and-effacing bacterial pathogens. Nat. Immunol.

[38] Basu R, O'Quinn DB, Silberger DJ, Schoeb TR, Fouser L, Ouyang W, Hatton RD (2012). Th22 cells are an important source of IL-22 for host protection against enteropathogenic bacteria. Immunity.

[39] Goto Y, Panea C, Nakato G, Cebula A, Lee C, Diez MG, Laufer TM (2014). Segmented filamentous bacteria antigens presented by intestinal dendritic cells drive mucosal Th17 cell differentiation. Immunity.

[40] Lécuyer E, Rakotobe S, Lengliné-Garnier H, Lebreton C, Picard M, Juste C, Fritzen R (2014). Segmented filamentous bacterium uses secondary and tertiary lymphoid tissues to induce gut IgA and specific T helper 17 cell responses. Immunity.

[41] Scott CL, Aumeunier AM, Mowat AM (2011). Intestinal CD103+ dendritic cells: master regulators of tolerance?. Trends Immunol.

[42] Coombes JL, Siddiqui KRR, Arancibia-Cárcamo CV, Hall J, Sun C-M, Belkaid Y, Powrie F (2007). A functionally specialized population of mucosal CD103+ DCs induces Foxp3+ regulatory T cells via a TGF-beta and retinoic acid-dependent mechanism. J. Exp. Med.

[43] Sun C-M, Hall JA, Blank RB, Bouladoux N, Oukka M, Mora JR, Belkaid Y (2007). Small intestine lamina propria dendritic cells promote de novo generation of Foxp3 T reg cells via retinoic acid. J. Exp. Med.

[44] Matteoli G, Mazzini E, Iliev ID, Mileti E, Fallarino F, Puccetti P, Chieppa M (2010). Gut CD103+ dendritic cells express indoleamine 2,3-dioxygenase which influences T regulatory/T effector cell balance and oral tolerance induction. Gut.

[45] Raymond M, Van VQ, Rubio M, Welzenbach K, Sarfati M (2010). Targeting SIRP-α protects from type 2-driven allergic airway inflammation. Eur. J. Immunol.

[46] Schulz O, Jaensson E, Persson EK, Liu X, Worbs T, Agace WW, Pabst O (2009). Intestinal CD103+, but not CX3CR1+, antigen sampling cells migrate in lymph and serve classical dendritic cell functions. J. Exp. Med.

[47] Takai S, Yamada M, Araki T, Koshimizu H, Nawa H, Hatanaka H (2002). Shp-2 positively regulates brain-derived neurotrophic factor-promoted survival of cultured ventral mesencephalic dopaminergic neurons through a brain immunoglobulin-like molecule with tyrosine-based activation motifs/Shp substrate-1. J. Neurochem.

[48] Lindberg FP, Bullard DC, Caver TE, Gresham HD, Beaudet AL, Brown EJ (1996). Decreased resistance to bacterial infection and granulocyte defects in IAP-deficient mice. Science.

[49] Bain CC, Mowat AM (2012). CD200 receptor and macrophage function in the intestine. Immunobiology.

