# Supplementary material for: Signal regulatory protein alpha (SIRPα) regulates the homeostasis of CD103+CD11b+ DCs in the intestinal lamina propria
Source: Eur J Immunol. 2014 Oct 27;44(12):3658–68. doi: 10.1002/eji.201444859 (PMC4284040; doi:10.1002/eji.201444859)
Supplement: Supplementary file 1 — Figure S1. Effect of SIRPα mutation on mononuclear phagocytes in the colonic lamina propria. Figure S2. Reduced population of CD103+CD11b+ DCs does not impact TReg cells in the colonic LP or affect the induction of oral tolerance. [file eji0044-3658-sd1.pdf]

European Journal of Immunology

Supporting Information

for

DOI 10.1002/eji.201444859

Charlotte L. Scott, Zangerle Murray TFP, Katherine S. H. Beckham, Gillian Douce and Allan McI. Mowat

Signal regulatory protein alpha (SIRPα) regulates the homeostasis of CD103$^+$CD11b$^+$ DCs in the intestinal lamina propria

© 2014 The Authors. *European Journal of Immunology* published by WILEY-VCH Verlag GmbH & Co. KGaA Weinheim

European Journal of Immunology

Supporting Information
for
DOI 10.1002/eji.201444859

Charlotte L. Scott, Zangerle Murray TFP, Katherine S. H. Beckham, Gillian Douce and Allan McI. Mowat

Signal regulatory protein alpha (SIRPα) regulates the homeostasis of CD103^{+}CD11b^{+} DCs in the intestinal lamina propria

© 2013 The Authors. European Journal of Immunology published by Wiley-VCH Verlag GmbH & Co. KGaA Weinheim

Supporting Information

Supporting Figure 1

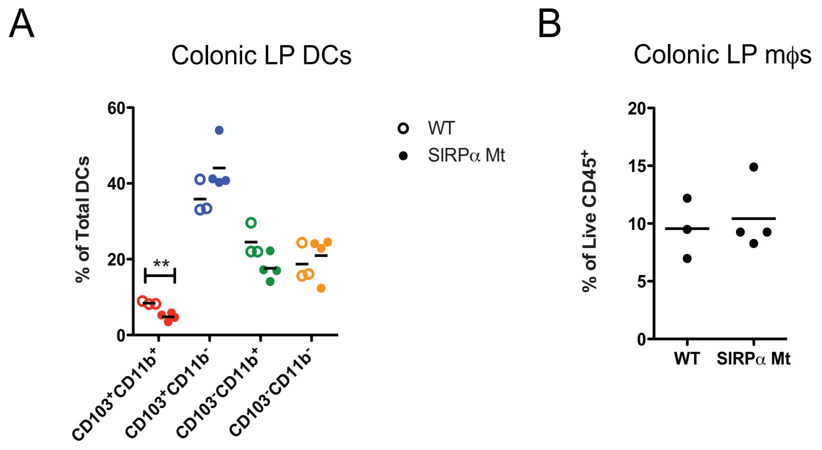

Supporting Figure 1: Effect of SIRPα mutation on mononuclear phagocytes in the colonic lamina propria.

A. Proportions of CD103/CD11b based subsets amongst live $CD45^+CD11c^+MHCII^+CD64^-B220^-$ DCs from colonic LP of SIRPα mt and WT mice. **B.** Proportions of $CD64^+$ macrophages amongst live $CD45^+CD11c^+MHCII^+$ cells in colonic lamina propria of SIRPα mt and WT mice. Data are representative of at least 3 independent experiments with n=3/4 per experiment. *p<0.05, **p<0.01, ***p<0.005 Student's t test.

Supporting Figure 2

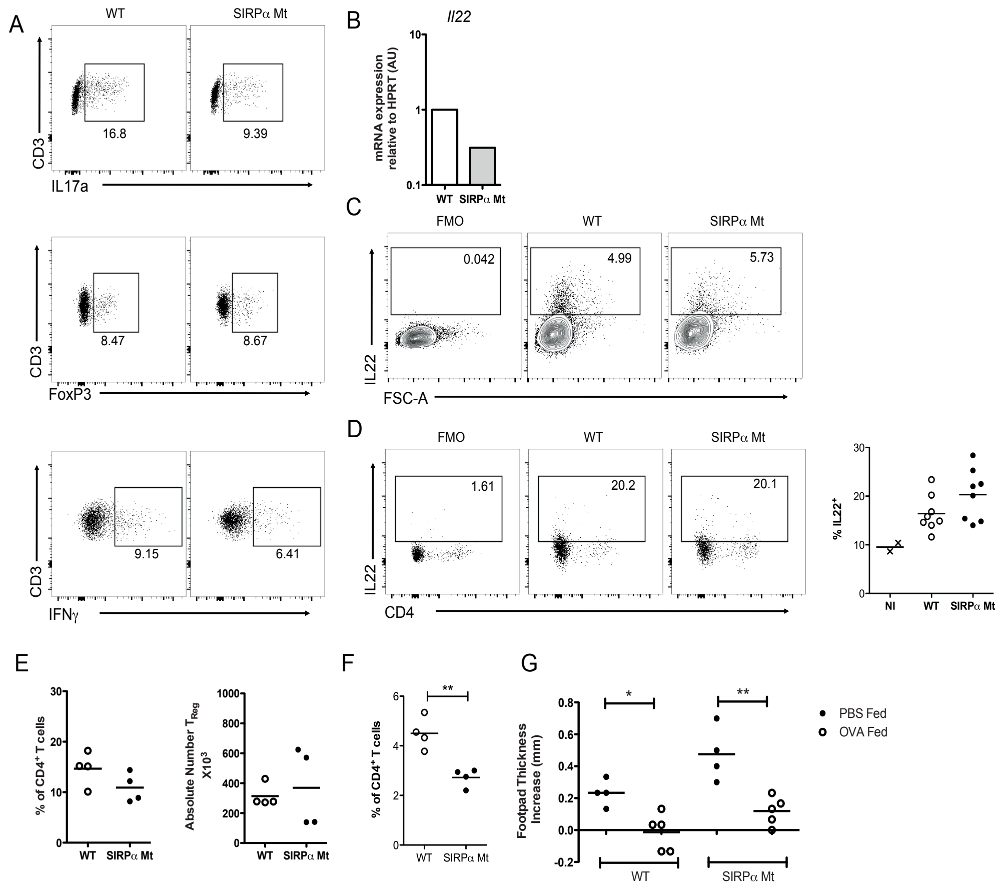

Supporting Figure 2: Reduced population of $CD103^{+}CD11b^{+}$ DCs does not impact T_{Reg} cells in the colonic LP or affect the induction of oral tolerance.

A. Representative Intracellular FACS staining for IL17a, FoxP3 and IFNγ by total $CD3^{+}CD4^{+}$ T cells from the SI LP of WT and SIRPα mt mice. **B.** Q-PCR analysis of mRNA for *Il22* by total $CD4^{+}$ T cells FACS-purified from WT or SIRPα mt SI LP. Data are from two experiments with 4 mice pooled and are expressed relative to HPRT using the $2^{-\Delta\Delta ct}$ method with WT set to 1**. C**. Representative staining of IL22 producing $CD4^{+}$ T cells in the colonic LP of

WT and SIRPα mt mice infected with $1x10^9$ *Citrobacter rodentium* 8 days before, assessed by flow cytometry. **D**. WT and SIRPα mt mice were infected with $1x10^9$ *C. rodentium* and on day 8 of infection colonic LP was examined for IL22-producing ILC3s (gated as live, $CD45^+CD3^-CD19^-NKp46^+RORgt^+$). Dot plots shows representative staining for IL22 from ILC3s and scatterplot shows proportion of $IL22^+$ cells as a % of total colonic ILC3s. Data are from 1 experiment with n=8. **E.** Proportions and absolute numbers of cells staining intracellularly for FoxP3 amongst total live $CD4^+$ T cells in the colonic lamina propria of SIRPα mt and WT mice. Data are representative of 2 independent experiments with n=4 per experiment. **F.** Proportions of cells staining intracellularly for FoxP3 amongst total live $CD4^+$ T cells in the mesenteric lymph node of SIRPα mt and WT mice. Data are from a single experiment with n=4. **G.** WT or SIRPα mt mice were fed a single dose of 25mg OVA or PBS and immunized in the right hind footpad with OVA emulsified in CFA 7 days later. 21 days later, mice were challenged in the contralateral footpad with HAO and 24 hours later, OVA specific DTH responses were assessed by measuring the increase in footpad size. OVA specific DTH responses are shown as the mean increase in footpad measurement of 4-5 mice per group and are representative of two independent experiments. *$p<0.05$, **$p<0.01$. One Way ANOVA with Bonferroni post-test.
